# Breathing Chest Wall Kinematics Assessment through a Single Digital Camera: A Feasibility Study

**DOI:** 10.3390/s23156960

**Published:** 2023-08-05

**Authors:** Nunzia Molinaro, Emiliano Schena, Sergio Silvestri, Carlo Massaroni

**Affiliations:** Unit of Measurements and Biomedical Instrumentation, Università Campus Bio-Medico di Roma, 00128 Rome, Italy; n.molinaro@unicampus.it (N.M.); e.schena@unicampus.it (E.S.); c.massaroni@unicampus.it (C.M.)

**Keywords:** chest wall kinematics, respiratory monitoring, thoraco-abdominal displacements, digital cameras

## Abstract

The identification of respiratory patterns based on the movement of the chest wall can assist in monitoring an individual’s health status, particularly those with neuromuscular disorders, such as hemiplegia and Duchenne muscular dystrophy. Thoraco-abdominal asynchrony (TAA) refers to the lack of coordination between the rib cage and abdominal movements, characterized by a time delay in their expansion. Motion capture systems, like optoelectronic plethysmography (OEP), are commonly employed to assess these asynchronous movements. However, alternative technologies able to capture chest wall movements without physical contact, such as RGB digital cameras and time-of-flight digital cameras, can also be utilized due to their accessibility, affordability, and non-invasive nature. This study explores the possibility of using a single RGB digital camera to record the kinematics of the thoracic and abdominal regions by placing four non-reflective markers on the torso. In order to choose the positions of these markers, we previously investigated the movements of 89 chest wall landmarks using OEP. Laboratory tests and volunteer experiments were conducted to assess the viability of the proposed system in capturing the kinematics of the chest wall and estimating various time-related respiratory parameters (i.e., *f_R_*, *T_i_*, *T_e_*, and *T_tot_*) as well as TAA indexes. The results demonstrate a high level of agreement between the detected chest wall kinematics and the reference data. Furthermore, the system shows promising potential in estimating time-related respiratory parameters and identifying phase shifts indicative of TAA, thus suggesting its feasibility in detecting abnormal chest wall movements without physical contact with a single RGB camera.

## 1. Introduction

Respiratory pattern detection and monitoring is a widely investigated topic not only in the clinical practice, focusing on the detection of respiratory-related pathologies [1,2,3], but also in scenarios like sports science [4,5]. During respiratory activity, thoracic and abdominal movements are coordinated since the diaphragm affects both the thoracic and the abdominal cavities [6]. In normal respiration, diaphragmatic breathing patterns (i.e., deep breathing, which relies on the movement of the diaphragm) and costal breathing patterns (i.e., shallow breathing based on the excursion of the rib cage) are involved. Lesions of respiratory centers in the pons and medulla oblongata, use of narcotic drugs, metabolic alterations, and respiratory muscle weakness can produce abnormal respiration patterns [7]. Among these, thoraco-abdominal movements are widely investigated since it is clinically observed in many respiratory disorders and/or respiratory muscle dysfunction and clinically assessed as a sign of respiratory stress and increased work of breathing [8].

The asynchronous movement of the thorax and the abdomen is referred to as thoraco-abdominal asynchrony (TAA) and is characterized by a time lag between the two compartments’ expansion. Pure paradoxical movement occurs when the thorax and the abdomen move simultaneously in completely opposite directions [8]. Mainly, TAA is related to some respiratory and neuromuscular disorders (e.g., Chronic Obstructive Pulmonary Disease, hemiplegia, Duchenne muscular dystrophy) [9,10,11]. However, evaluating thoraco-abdominal kinematics plays an important role in developing specific training and improving breathing mechanics and performance in sport science [12].

Investigating thoraco-abdominal kinematics can be of particular interest to identify differences between thoracic and abdominal respiration, allowing for the assessment of motions of the thorax and abdomen. Phase angle (PA) and phase shift (PS) are the most used parameters to quantify TAA. To compute PA, the thoracic and abdominal signals are plotted against each other (i.e., Lissajous figure), and the opening angle of the figure indicates the level of asynchrony between the two compartments [8,9]. PS is related to paradoxical motion and represents the percentage of the respiratory cycle, in which the compartments move in opposite directions [9]. In addition, since variations in the coordination of muscle contraction are involved in dysfunctional breathing and sensations of dyspnea [13], the continuous monitoring of respiratory-related parameters (e.g., respiratory frequency (*f_R_*), inspiratory time (*T_i_*), and expiratory time (*T_e_*)) may be crucial for detecting respiratory disorders, such as apnea and thoraco-abdominal asynchronies [7]. The authors in [6] investigated the coordination of thoraco-abdominal movements depending on *f_R_* to evaluate the effect of different *f_R_* on thoraco-abdominal kinematics.

Contact-based or contactless technologies can independently detect breathing patterns and thoraco-abdominal motions. Among the contact-based techniques, wearable systems based on different types of sensors (e.g., piezoresistive, capacitive, and fiber optic sensors) and their appropriate placement on the subject’s torso have been employed [14,15]. Respiratory inductance plethysmography (RIP) is the most used contact-based system to quantify chest wall movements. It involves using two belts, one thoracic and one abdominal, surrounding the subject’s torso [16]. Although the results are promising, these methods may create discomfort to the subject, are prone to influence their breathing, and in some cases, they can be simply inconvenient or impractical.

In recent years, non-contact systems for chest wall movements detection have been explored, including motion capture systems, like optoelectronic plethysmography (OEP) [17,18] and depth cameras (e.g., structured light, time-of-flight) [19,20,21]. OEP is mainly used, and it is considered the gold standard for evaluating chest wall motions and detecting respiratory volumes. In both cases, these technologies require time-consuming and extensive processing of data in order to obtain the information of interest. In this scenario, digital cameras are gaining much interest thanks to their ease of availability, non-invasiveness, and low cost for detecting respiratory activity by framing the upper body without any contact with the subject and evaluating respiratory-related parameters [21,22,23,24,25]. However, it is worth noting that there have been limited research studies that have utilized digital cameras, including RGB cameras found in smartphones, PCs, and professional cameras [26,27,28,29], to capture thoraco-abdominal movements [22,30]. These studies mainly focus on assessing the use of digital cameras in the detection of chest wall movements to estimate respiratory-related parameters (e.g., *f_R_*). For example, Reyes et al. proposed a smartphone-based system for the detection of both chest wall movement and respiratory sounds for the classification of inspiratory and expiratory phases [29]. The authors in [26] compared the breathing waveforms obtained from three video-based methods (i.e., RGB, depth, and thermal cameras) with the aim of characterizing the differences in the achieved respiratory rhythm. Surprisingly, there has been a lack of comprehensive analysis on the effectiveness of these cameras in monitoring thoraco-abdominal asynchrony (TAA).

In this paper, we aim to investigate the feasibility of using a single RGB digital camera for capturing thoracic and abdominal displacements with non-reflective markers placed on the torso to derive information about chest wall kinematics and TAA. The major contributions of our paper relies on (1) a comprehensive analysis of chest wall deformation performed using OEP system to identify specific areas that exhibit substantial rib cage displacement during breathing, which can serve as optimal points for attaching non-reflective markers, (2) the design of a measuring system based on a single calibrated RGB digital camera and its performance assessment in the laboratory environment, and (3) a explorative investigation of TAA by computing both the phase shift between the thoracic and the abdominal movements and time-related respiratory parameters (e.g., *f_R_*, *T_i_*, *T_e_*, and *T_tot_*) on volunteers.

## 2. Analysis of Chest Wall Displacements

The proposed non-contact system comprises a hardware module (i.e., a single digital camera) and non-reflective markers placed on the torso of the subject. Before describing the proposed system, the prior analysis is explained in the following sections to identify the regions of the torso that express the most significant displacements. In detail, the proposed method involves (1) the calibration of the single digital camera, (2) the implementation of an algorithm to automatically identify non-reflective markers and getting markers displacements in mm, and (3) the implementation of algorithms to estimate time-related respiratory parameters based on a breath-by-breath analysis as well as TAA indexes.

### 2.1. Analysis of Markers’ Displacements

OEP is the gold standard for recording the chest wall kinematics (and 3D displacement) and for calculating the chest wall volume variations (both global and compartmental). OEP consists of a minimum of four IR cameras that record the 3D trajectories over time of a number of IR photo-reflective markers placed on the torso of the subject. The number of cameras, their calibration, and the marker protocols strongly influence the accuracy in the 3D position measurement and the overall volume calculation [31,32].

As a first stage of our study, we carried out the chest wall displacements analysis using OEP to (1) identify the magnitude of the displacements to which the rib cage is exposed during breathing, (2) evaluate the axis along which displacements are prevalent, and (3) identify the best position to place the non-reflective markers, which is necessary for carrying out the measurement with the proposed system.

Data collected from ten healthy volunteers (i.e., ten males, age range 19–37 years old, height between 163 cm and 193 cm, and body mass between 62 kg and 93 kg) were analyzed. All the tests were carried out in compliance with the Ethical Approvals (ST-UCBM 27/18 OSS). An OEP system (BTS D-Smart, produced by BTS Bio-Engineering S.r.l., Milan, Italy) was used. It consists of eight IR cameras arranged in a circle so that the volunteer is in the center of the scene. The trajectories of 89 hemispherical photo-reflective passive markers placed on the participant’s chest wall (42 on the anterior surface, 37 on the posterior wall, and ten on the lateral zone) were collected with a sampling rate of 60 Hz using the tracker software provided by BTS (BTS, Bioengineering S.r.l., Milan, Italy). During the test, participants in a standing position were asked to initially breathe quietly, hold their breath for ~5 s and then continue breathing quietly for ~120 s.

The 3D trajectories of the markers collected with the OEP system were analyzed to identify the magnitude of the displacements and the regions of the torso that move most during the breathing activity.

#### 2.1.1. Calculation of the Chest Wall Volume from the OEP System

The chest wall global volume (*V_TOT_*) was obtained by analyzing the markers’ data. In particular, the geometric model presented in [18] was used to obtain the chest wall volume from the 3D marker coordinates. The prism-based method was used to compute the volume of each of the 82 prims at each frame. Considering *P_1,i_*, *P_2,i_*, *P_3,i_*, and *P_4,i_*,the vertices of the *i*-th tetrahedron and the *i*-th volume (*V_i_*) can be obtained through Equation (1):

(1) $$V_{i}=\frac{1}{6}\left|det(V_{1,i},\;V_{2,i},\;V_{3,i})\right|=\frac{1}{6}\left|\det\left[\begin{array}{cccc} 1 & x_{P1} & y_{P1} & z_{P1}\\ 1 & x_{P2} & y_{P2} & z_{P2}\\ 1 & x_{P3} & y_{P3} & z_{P3}\\ 1 & x_{P4} & y_{P4} & z_{P4} \end{array}\right]\right|$$

where *V_1,i_* = *P_2,i_* − *P_1,i_*, *V_2,i_* = *P_3,i_* − *P_2,i_*, and *V_3,i_* = *P_4,i_* − *P_3,i_*. *V_TOT_* was then computed by summing the volumes of all the tetrahedrons.

#### 2.1.2. Identification of Magnitude of the Displacements and Best Axis Selection

**Figure 1:**
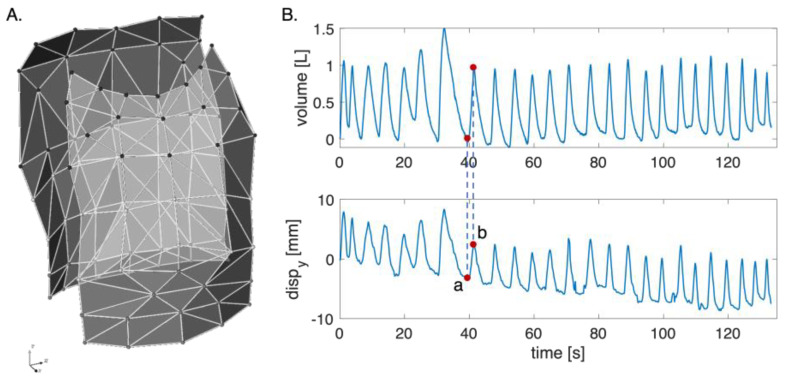
(**A**) Schematic representation of the chest wall with the orientation of the three axes (i.e., *x*, *y*, and *z*). (**B**) Volume and displacements signal along the *y*-axis per one marker: (a) represents the $coord_{r}\left({loc}_{min,i}\right)$ corresponding to the minimum point on the volume signal; and (b) is the $coord_{r}\left({loc}_{max,i}\right)$ corresponding to the maximum point on the volume signal.

Each photo-reflective marker is described by three coordinates—*x*, *y*, *z*—in the space (Figure 1A). Since the proposed system is based on a single digital camera framing the anterior surface of the torso, only the trajectories of the 42 frontal markers were analyzed. The apnea stage was not considered in the analysis since the computed volume and the trajectories were cut starting from the first minimum point after the apnea.

To reconstruct the markers’ displacement, each respiratory act was identified based on the volume by selecting the minimum point corresponding to the end of inspiration and the maximum point of the start of inspiration. Each respiratory act along the coordinates *x*, *y*, and *z* was obtained according to Equation (2):

(2) $$displacement_{r}=coord_{r}\left({loc}_{max,i}\right)-coord_{r}({loc}_{min,i})$$

where $displacement_{r}$ represents the displacement of the marker along the *x-*, *y-,* and *z*-axes; $coord_{r}\left({loc}_{max,i}\right)$ is the marker coordinate at the maximum point identified on the volume signal per axis; and $coord_{r}\left({loc}_{min,i}\right)$ is the marker coordinate at the minimum point identified on the volume signal per axis (see Figure 1B).

Then, for each subject, the mean value of the displacement along each axis was computed, and an uncertainty analysis was carried out to identify the magnitude of the displacements and the axis on which the displacements are prevalent. The uncertainty was computed as reported in Equation (3):

(3) $$\delta x=k\cdot \frac{S_{x}}{\sqrt{N}}$$

where *k* is the coverage factor, *S_x_* is the standard deviation, and *N* represents the number of measurements. According to *N*, the cover factor was estimated with the Gaussian distribution if *N* > 30 or with the Student distribution if *N* ≤ 30 [33].

**Figure 2:**
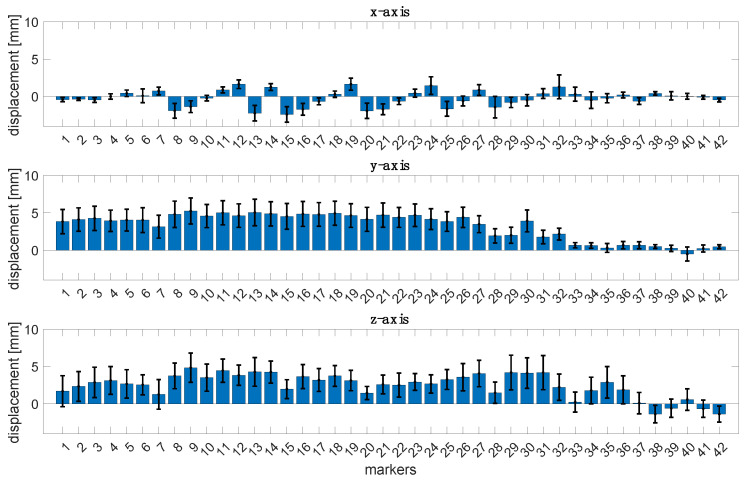
Bar plots of the mean displacements of the 42 markers along the *x-*, *y-*, and *z*-axes with the corresponding uncertainty for all the subjects.

Figure 2 reports the bar plots regarding the mean displacements along the *x-*, *y-*, and *z*-axes with the corresponding uncertainty of the 42 markers considering all the subjects. Results show that the axis where the displacements are widespread is the *y*-axis, whereas the magnitude displacements are less than 10 mm.

#### 2.1.3. Identification of the Best Position of the Markers: Principal Component Analysis

**Figure 3:**
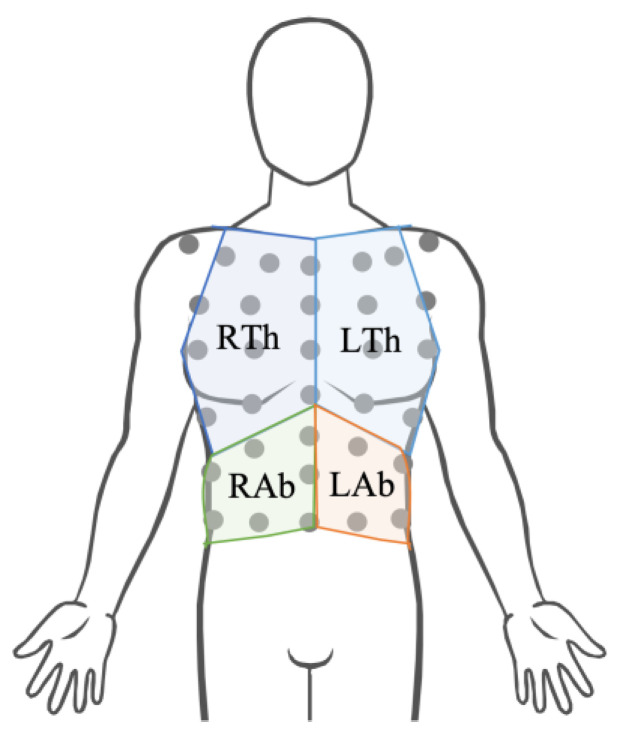
Representation of the four compartments of the chest wall (i.e., right thorax—RTh, right abdomen—Rab, left thorax—LTh, and left abdomen—Lab).

To identify the markers that showed the most significant displacement during respiratory activity considering the whole volunteer population, we used Principal Component Analysis (PCA), one of the most used methods for dimensionality reduction. It allows for the representation of the observed signals as a set of new orthogonal variables defined as Principal Components (*PCs*) [34]. Given the results depicted in Figure 2, we considered only the displacements along the *y*-axis of the 42 markers partitioned into the four compartments of the chest wall (right thorax—RTh, right abdomen—Rab, left thorax—LTh, and left abdomen—Lab), as shown in Figure 3.

Before applying PCA, the signals were detrended to remove the mean value and the noise. Based on the method presented in [35], we determined which markers expressed the most significant displacement per compartment by performing the following steps:

- The *p* components with an accounted variance equal to 95% were preserved.
- The weight of the *i*-th marker (*w_i_*) along the *p PCs* were computed as follows:

(4) $$\left\{\begin{array}{c} w_{i}=\frac{z_{i}}{\sum_{i=1}^{N}z_{i}}\cdot 100\;[\%]\\ z_{i}=\sum_{k=1}^{p}\left|u_{i,k}\right| \end{array}\right.$$

- In Equation (4), $\left|u_{i,k}\right|$ represents the absolute value of the elements of the matrix **U** related to the *i*-th marker and the *k*-th *PC* [35].
- The obtained values of percentage weights per each compartment were evaluated, and the markers which express the most significant displacement were identified. This information is fundamental to determine which parts of the anterior surface of the torso move the most since monitoring only those regions by applying non-reflective markers and the proposed system is required.

The mean percentage weight ($w_{mean}^{i}$) expressed by each marker was computed for each of the four compartments, as reported in Equation (5):

(5) $$w_{mean}^{i}=\frac{\sum_{i=1}^{N}w_{i}}{N}$$

where $w_{i}$ is the weight of the *i*-th marker, and *N* represents the number of subjects.

**Figure 4:**
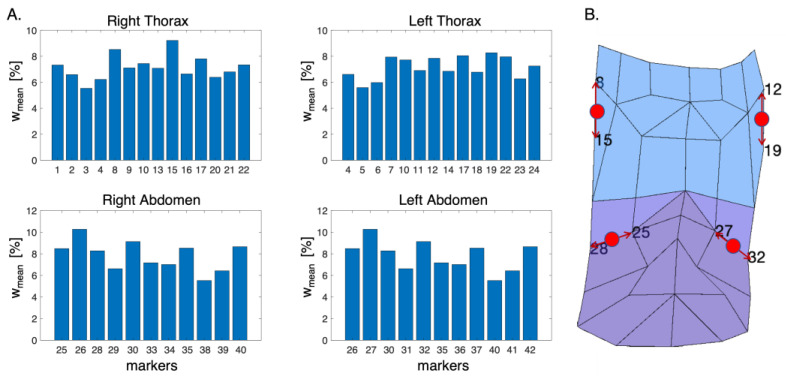
(**A**) Bar plots of the mean percentage weight expressed by each marker for each compartment. (**B**) Schematic representation of the anterior chest wall with highlighted the positions of the non-reflective markers (in red). The red arrows identify the photo-reflective markers used as reference to place the non-reflective ones.

In Figure 4A, the bar plots representing the mean percentage weight for each compartment are reported. The markers which express the highest mean percentage weight represent the ones that moved the most during the respiratory activity.

Based on the obtained results, the suitable positions on the torso’s subject for the apposition of the non-reflective markers were identified, as shown in Figure 4B (i.e., between markers 8 and 15 for RTh, between markers 12 and 19 for Lth, between markers 25 and 28 for Rab, and between markers 27 and 32 for Lab).

## 3. Proposed Contactless System

### 3.1. Calibration Procedure of the Digital Camera

To estimate the 3D markers’ trajectories from a video recorded with a single digital camera, we need to calibrate the device to extract metric information from 2D images. The calibration procedure presented in [34] was used for this aim. The proposed method allows for the retrieval of the calibration parameters by implementing the following steps:

- Print a pattern and attach it to a planar surface. The most used pattern is a checkerboard, which should include an even number of squares along the *y*-axis and an odd number along the *x*-axis.
- Take a few images of the model plane under different orientations by moving the plane or the camera (at least ten images).
- Detect the feature points in the images.
- Estimate the intrinsic and extrinsic parameters.

The relationship between a 3D point M and its image projection m is given by Equation (6):

(6) $$s\cdot \tilde{m}=A[R\;t]\tilde{M}$$

where *s* is an arbitrary scale factor; (***R***, ***t***) shows that the extrinsic parameters are the rotation and the translation matrixes, which relate the world coordinate system to the camera coordinate system; and ***A*** is the camera intrinsic matrix, which includes the focal length, the optical center, and the skew coefficients [36].

The calibration procedure was performed in a MATLAB environment. The pattern used for the calibration was a checkerboard, which was fixed on a planar surface, thereby avoiding possible irregularities that could affect the accuracy of the calibration. The acquisition of 10 images in different orientations was carried out by positioning the calibration pattern on a uniform background to avoid interfering factors that can influence the right acquisition of the calibration pattern.

**Figure 5:**
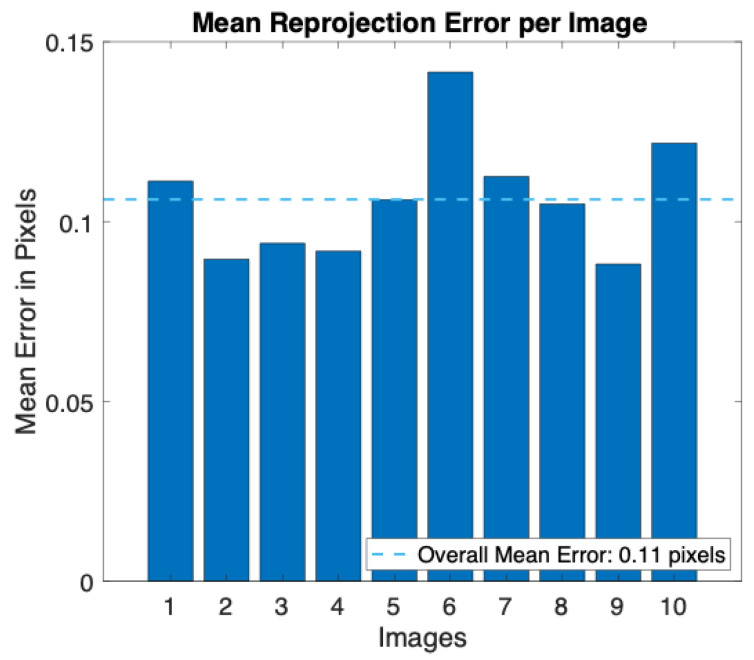
Bar plot of the mean reprojection error per image obtained during the calibration procedure.

A smartphone’s built-in digital camera (i.e., iPhone 8 Plus, Apple Inc., Cupertino, CA, USA) was calibrated following the steps described above. The images were acquired with a resolution of 720 × 1280 pixels, the same as will be used for video recording, and the camera was placed at about 70 cm from the calibration pattern. During the acquisition all the camera parameters were fixed (e.g., ISO and shutter speed), and the autofocus was locked. After image acquisition, all the calibration parameters were retrieved. In Figure 5, we report the reprojection errors, which provide a qualitative measure of the accuracy of the calibration. The reprojection error is the distance between a pattern key point (i.e., a point detected in the checkerboard) detected in a calibration image and a corresponding world point projected into the same image. An overall mean error of 0.11 pixels was achieved during the calibration.

### 3.2. Estimation of Displacements from Video Recorded with a Digital Camera: Laboratory Assessment

To evaluate the performance in the estimation of marker trajectories from video recorded with a single digital camera, tests in the laboratory were performed. The displacement of four non-reflective circular markers with different diameters (i.e., 24 mm, 22 mm, 20 mm, and 18 mm, named marker 1, marker 2, marker 3, and marker 4, respectively) on a monitor was simulated. These markers were specially designed and colored bright pink to simplify their identification during video analysis.

The calibrated smartphone’s built-in digital camera (i.e., iPhone 8 Plus, Apple Inc., Cupertino, CA, USA) was used to record videos with a resolution of 1280 × 720 pixels and an acquisition frequency of 30 frame per second (*fps*). The distance between the camera and the monitor is about 70 cm. Four displacements (i.e., 101 mm, 51 mm, 21 mm, 11 mm) were set for the markers to cover over three time intervals (i.e., 3 s, 1.5 s, 1 s). These parameters were set to simulate different stages of the breathing activity (i.e., quiet breathing, deep inspiration, and deep expiration). A video for each displacement at each time interval was recorded. During the recording, each marker reached the fixed displacement (i.e., reference displacement) and returned to the initial position, with the movement repeating ten times.

During the video processing, for each video frame, the four markers were identified and sorted in descending order according to the area. For each marker, (i) the center, (ii) the distance between the marker’s center and the camera, and (iii) the diameter were identified. Then, the calibration parameters obtained from the calibration procedure were used to convert the identified 2D coordinates from pixels to mm. The displacements along the *x-*, *y-*, and *z*-axes are represented by the displacements of the marker center (along the *x-* and *y*-axes) and the marker center–camera distance (along the *z*-axis).

To evaluate the performance of the non-contact system in estimating the displacements expressed by the four markers, the error was calculated as the difference between the estimated displacement and the reference one. In addition, the Mean Percentage Absolute Error (MAPE) was computed for each marker and the mean value of the MAPE with the relative uncertainty was calculated to evaluate how the error changes independently from the dimension of the markers and the covered displacements.

**Figure 6:**
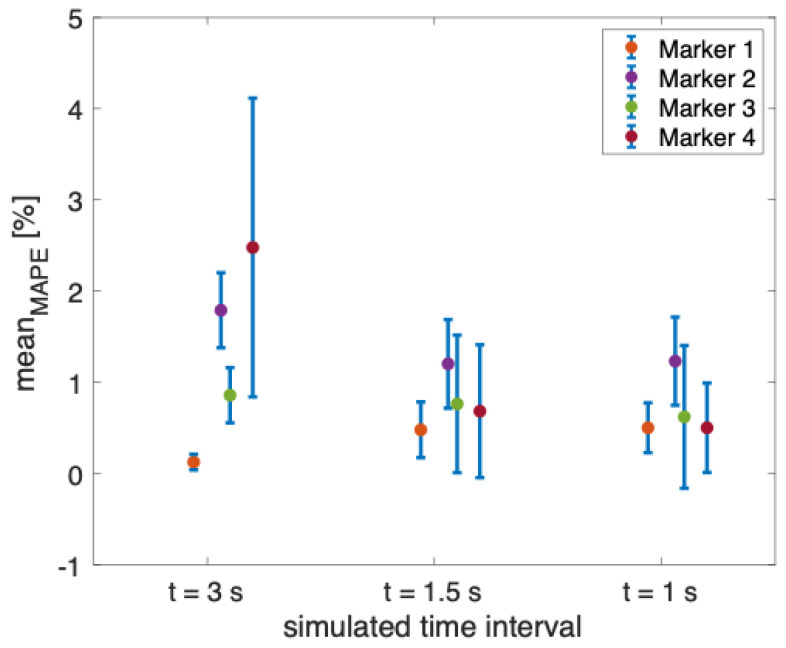
Mean value of the MAPE in percentage for each non-reflective marker during the three simulated time intervals (i.e., t = 3 s, t = 1.5 s, and t = 1 s).

The results showed that the error in estimating the displacements is always lower than 1.50 mm, independent of the markers’ diameter for all the performed tests during the three simulated time intervals (see Table 1). However, the error increases with the covered distance (e.g., an error greater than 1 mm was obtained when the covered distance is 101 mm). When considering a covered distance of 11 mm, a displacement comparable to that of the chest wall during quiet breathing [37], and the error is close to 0 mm in all the performed tests. In addition, as can be noted from Figure 6, the mean values of the MAPE for all the markers are always below 2.47%, with the highest values of uncertainty obtained from marker 4 when simulating the 3 s time interval (i.e., δx = 1.64%).

Based on the promising results obtained during the laboratory assessment, we tested the proposed method on healthy volunteers to evaluate the feasibility of use a calibrated digital system for the measurement of breathing kinematic variables.

## 4. Tests on Healthy Volunteers

Tests were performed on four healthy male volunteers (aged between 24 and 34 years old, with body mass between 61 kg and 88 kg and height between 163 cm and 187 cm). The study was conducted according to the guidelines of the Declaration of Helsinki. Informed consent was obtained from all subjects involved in the study (ST-UCBM 14/22 OSS).

**Figure 7:**
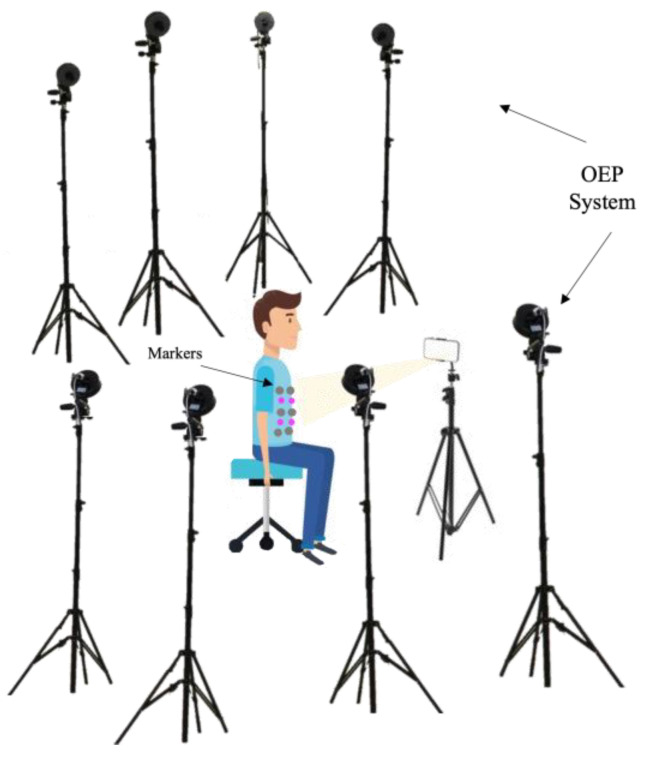
Experimental setup composed of eight cameras of the OEP system, the photo-reflective markers, the iPhone 8 camera, and the four non-reflective markers.

Each volunteer was asked to sit on a chair without a backrest facing the digital camera, remaining as motionless as possible. The tests were performed in the laboratory with an eight-camera OEP system used as a reference (as the previous described). The proposed system includes a digital RGB camera and four non-reflective markers with different diameters (i.e., 24 mm, 22 mm, 20 mm, and 18 mm) attached on the subject’s torso at the level of the photo-reflective markers, which express the best displacements (see Section 2.1.3). The camera was placed on a tripod about 1 m from the subject. Before starting the tests, the digital camera was calibrated according to the process described in Section 2.1. The experimental setup is reported in Figure 7.

During the tests, each subject was asked to perform an initial apnea stage of ~15 s, followed by ~90 s of quiet breathing, an apnea of ~15 s, and ~90 s of deep inspiration and expiration.

### 4.1. Data Analysis

The collected videos were post-processed in a MATLAB environment to extract the trajectories of the four non-reflective markers attached to the subject’s torso, according to the steps described in Section 3.2. Respiratory traces from each performed trial were obtained: (i) quiet breathing—*d_QB_*, and (ii) deep inspiration and expiration—*d_IE_*. The respiratory reference patterns were obtained by computing the total volume according to the steps explained in Section 2.1.1. In addition, the four compartmental volumes were computed considering six tetrahedrons, according to the method reported in [18].

**Figure 8:**
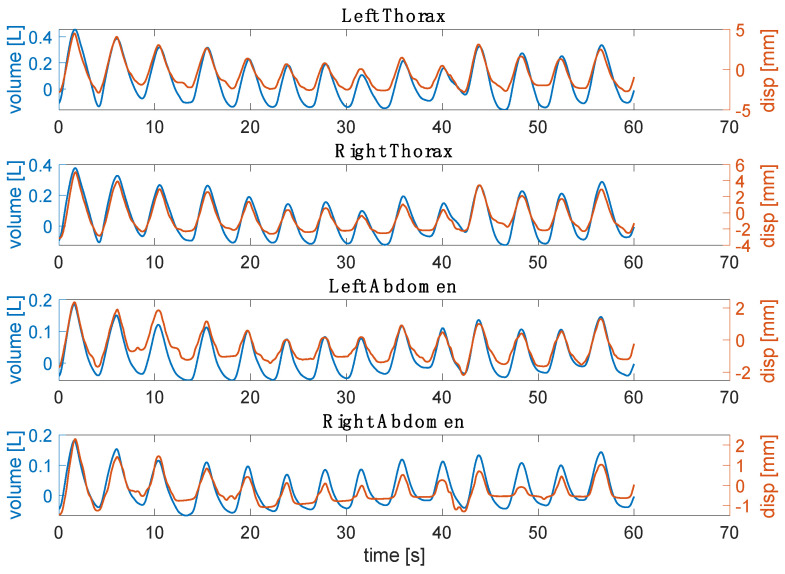
Example for one subject of the respiratory waveform retrieved from the video against the reference volume for each compartment.

The retrieved respiratory waveforms from videos and the reference were synchronized starting from the first minimum point after the initial apnea stage (Figure 8). Then, the signals were filtered with a bandpass filter between 0.01 Hz and 1 Hz. The first 60 s of the signals were used to compute the time-related respiratory parameters (i.e., *f_R_*, *T_i_*, *T_e_*, and *T_tot_*) [24]. In addition, an explorative assessment of thoraco-abdominal asynchrony was performed by computing the PA and PS [9].

**Figure 9:**
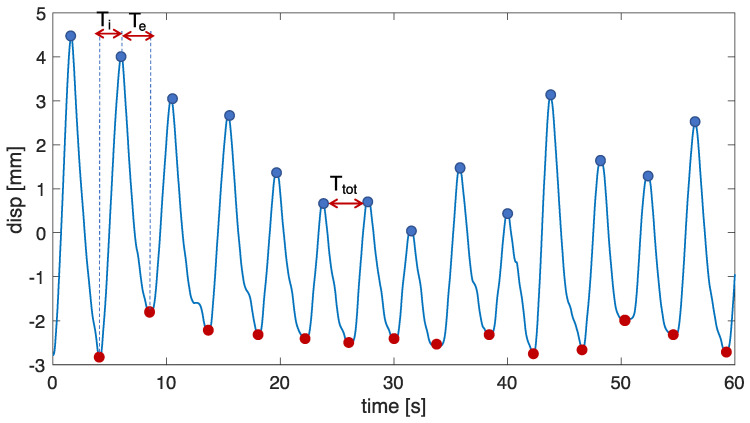
Scheme of the procedure adopted to extract temporal parameters from both the respiratory signals obtained from the non-contact system and the OEP. The procedure was performed for each compartment.

The time-related respiratory parameters were computed as follows:

- *f_R_* estimation: to extract breath-by-breath *f_R_* from V_TH_, V_AB_, *d_QB_*, and *d_IE_* for each performed trial (i.e., quiet breathing and deep IN/ES), the following steps were performed: (i) the duration of each breath (*T_tot_*) related to the *i*-th breath was retrieved as the time elapsed between two consecutive maximum peaks (expressed in s); and (ii) the related *i*-th *f_R_* was calculated as 60/*T_tot_* (expressed in breaths per minute (bpm)).
- Inspiratory, expiratory, and total time estimation: for each performed trial, *T_i_* was computed as the time difference between the time at which the maximum peak occurs and the time at which the minimum peak for the *i*-th breath occurs; *T_e_* was the time difference between the minimum peak and the time at which the maximum peak for the *i*-th breath occurs; *T_tot_* is the time difference between two consecutive maximum peaks (see Figure 9).

**Figure 10:**
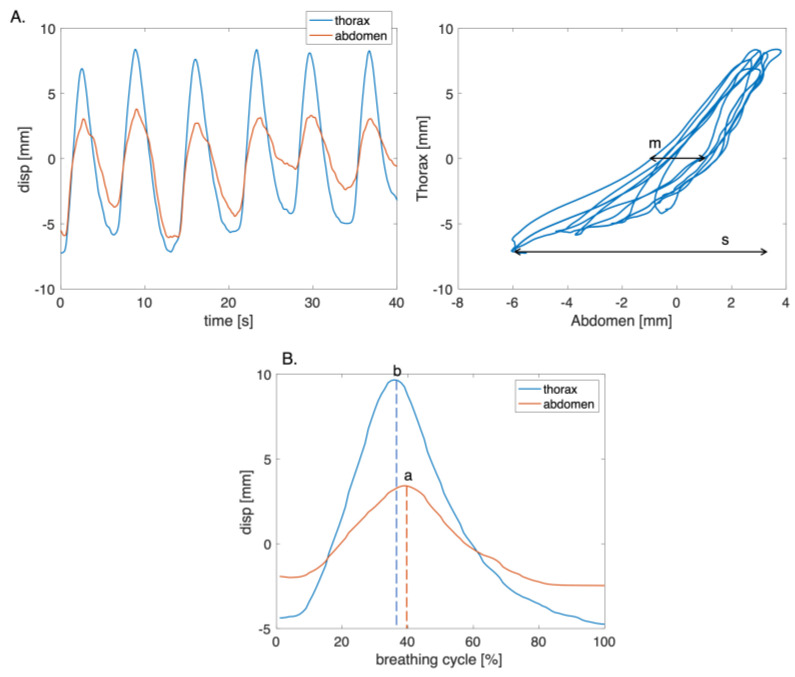
(**A**) Plot of the thoracic and abdominal compartments signals with the corresponding figure of Lissajous of the six respiratory acts, where *m* is the volume displaced by the abdomen at 50% of the thorax volume, while *s* represents the total volume displaced by the abdomen. (**B**) Example of a single respiratory act used to compute PS, where *a* and *b* are the time points at which the change in the direction of the thorax and the abdomen signals occur.

An explorative investigation of respiratory asynchronies was carried out considering only the thoracic and abdominal signals obtained in the deep phase since, during this exercise, the chest wall expresses movements that may be associated with respiratory asynchronies [38,39]. The Euclidean distances between markers 1 and 2, and between markers 3 and 4 were computed to obtain the thoracic and abdominal signals from videos, respectively. The PA (i.e., θ) and PS were computed considering six consecutive and homogeneous respiratory acts. For the computation of PA, the Lissajous figures were obtained by plotting the thoracic signal on the *y*-axis and the abdominal signal on the *x*-axis (Figure 10A). The PA was computed for each loop corresponding to a single respiratory act as reported in [40]:

(7) $$\theta_{i}=\sin^{-1}\left(\frac{m}{s}\right)$$

where *m* is the volume displaced by the abdomen at 50% of the thorax volume, while *s* represents the total volume displaced by the abdomen. θ was obtained for each volunteer as the mean value of $\theta_{i}$.

The PS parameter was computed by identifying the point at which there was a change in the direction of the signals during the respiratory cycle (Figure 10B). Subsequently, the time interval during which the compartments moved in opposite directions was calculated. The PS value was obtained as the ratio between the time interval and the total respiratory cycle time [9]. A linear transformation was used to express PS in terms of arc degree as reported in Equation (8):

(8) $$Phase\;Shift:\theta_{PS}=\frac{\left(b-a\right)}{\Delta t}\cdot 180$$

where *b* and *a* are the time points at which the change in the direction of the thorax and the abdomen signals occur, respectively, and Δ*t* represents the total time of the respiratory cycle. A positive angle indicates that the motion of the superior compartment is leading [9].

A Bland–Altman analysis was performed to assess the non-contact system’s performance in estimating the time-related respiratory parameters [41]. The obtained values of PA and PS were presented as the median and the interquartile range (IQR) for the reference and the estimated values.

### 4.2. Results

**Figure 11:**
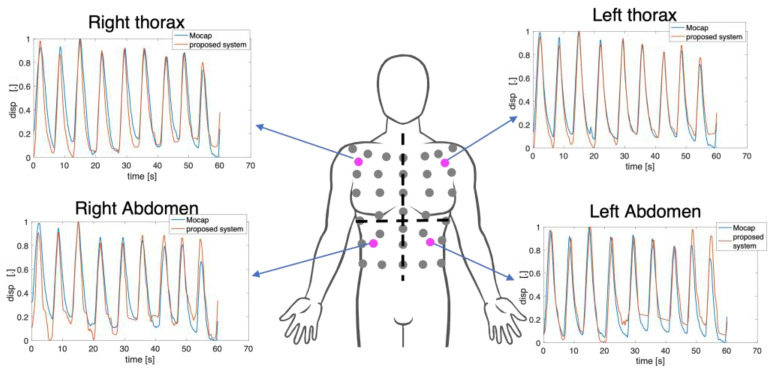
Normalized marker’s displacements extracted from the video against the reference displacements for each compartment per subject. In magenta are the non-reflective markers used in the proposed system.

Figure 11 reports the obtained displacements per compartment (i.e., RTh, RAb, LTh, and LAb) from four non-reflective markers attached to the torso of the subject against those estimated from the reference system. In detail, the displacements expressed by markers closest to the non-reflective ones were used. There is a good agreement between the signals extracted from the video and the reference one in terms of amplitude and trend both for the thoracic and the abdominal displacements.

**Figure 12:**
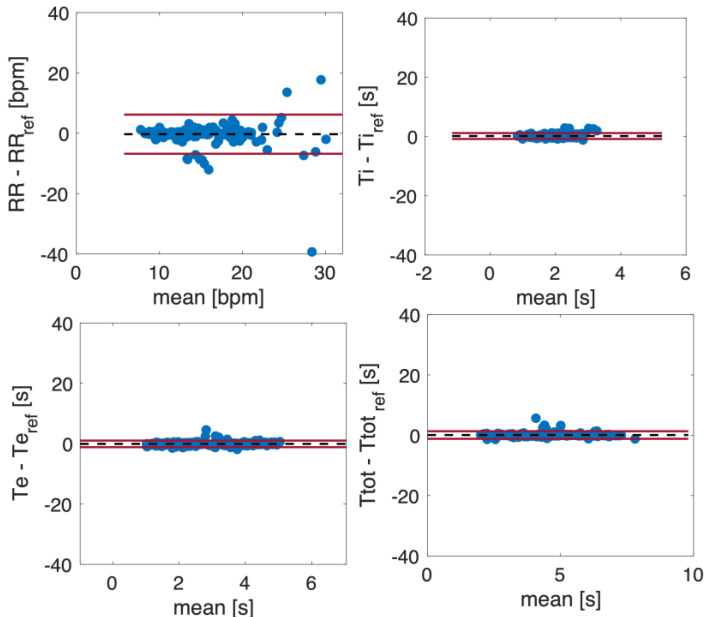
Bland–Altman plots comparing *f_R_*, *T_i_*, *T_e_*, and *T_tot_* estimated from respiratory signals obtained from a video.

Figure 12 shows the Bland–Altman plots for the *f_R_*, *T_i_*, *T_e_*, and *T_tot_* values estimated from the respiratory signals retrieved from the video. The dashed line represents the Mean of Difference (MOD), and the red lines are the upper and lower Limit of Agreement (LOA). The Bland–Altman plots include all the subjects’ values, considering quiet and deep breathing. Both *f_R_* and *T_tot_* estimated from the video signals show a good agreement with the reference ones, with a MOD ± LOAs of −0.3 ± 6.5 bpm and MOD ± LOAs = 0.08 ± 1.25 s, respectively. Considering *T_i_* and *T_e_*, LOAs below 1 s were achieved.

Table 2 reports the mean ± standard deviation (SD) obtained in the estimation of *f_R_*, *T_i_*, *T_e_*, and *T_tot_* for each volunteer. The mean values obtained for each parameter across all volunteers using the proposed non-contact system are quite comparable, indicating that the system provides accurate estimations of these parameters when compared to the reference values.

**Figure 13:**
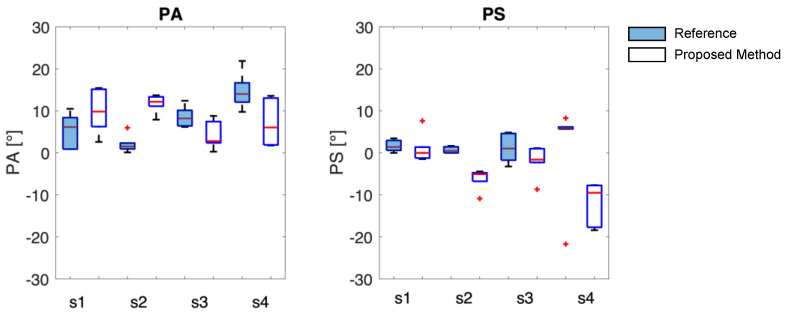
PA and PS results are presented as boxplots, where the center line is the median, and box limits indicate the 25th (lower limit) and 75th percentiles (upper limit). The lines above and below the box limits represent the largest and smallest values, respectively. The red + identify the outlier values.

The TAA indexes are reported with PA and PS values with boxplot where the median and the IQR (Figure 13) are shown to visually compare instruments in the estimation of the asynchrony between the thorax and the abdomen in healthy volunteers during deep respiration. The blue filled boxplot represents the results obtained with the reference system.

First and foremost, it is necessary to observe that the same data from the OEP system yield different results for thoraco-abdominal asynchrony (TAA) when assessed using the phase angle (PA) and the phase shift (PS), with maximum differences of up to 8° in median values. This behavior is consistent with the proposed system. Lower errors are observed in the median values when TAA is assessed using PA, with a maximum of 11° between the proposed method and OEP. The proposed system does not appear to systematically overestimate or underestimate the median values of TAA, when compared to reference values. It is worth noting that the TAA values never exceed 15° in median value, confirming that the enrolled subjects are healthy and have synchronous breathing between the thorax and abdomen.

## 5. Discussion and Conclusions

Evaluating thoraco-abdominal displacements and variations in time-related respiratory parameters may help monitor an individual’s health status and people suffering from neuromuscular disorders (e.g., hemiplegia and Duchenne muscular dystrophy).

In this study, a feasibility assessment of a single calibrated RGB digital camera for detecting thoracic and abdominal kinematics from non-reflective markers placed on the torso was performed. With this aim in mind, an initial analysis of the marker displacements in the OEP system was directed to identify the regions with the most pronounced rib cage movements. Subsequently, while using the findings from this analysis, tests were carried out on healthy volunteers. An exploratory examination of thoraco-abdominal asynchronies was performed by calculating both the phase shift between thoracic and abdominal movements and time-related respiratory parameters (e.g., *f_R_*, *T_i_*, *T_e_*, and *T_tot_*).

Results obtained from the initial analysis on the displacements of the OEP’s markers show that four regions on the anterior surface of the torso move most during respiratory activity. The following photo-reflective markers represent these regions: (i) markers 8 and 15 on the RTh, (ii) markers 12 and 19 on the LTh, (iii) markers 26 and 30 on the RAb, and (iv) markers 27 and 32 on the LAb. To the best of our knowledge, only one study [42] performed a similar analysis. Our results are in accordance with those reported in [42], in which the lateral regions of the torso were identified as the areas with the most significant movement. Based on these results, tests with a single RGB digital camera framing the anterior surface of the torso were performed. An evaluation of the displacements estimated from video acquired with a single calibrated digital camera was conducted. Displacements of the chest wall expressed in mm can be retrieved from a video with good accuracy. This suggests the potentiality of using such technology to assess and evaluate the chest wall biomechanics. Thus, these signals were used to estimate time-related respiratory parameters and respiratory asynchronies. Results show that the proposed non-contact system allows for the estimation of *f_R_* values and temporal parameters (i.e., *T_i_*, *T_e_*, and *T_tot_*) that are comparable with those of wearable systems (e.g., a bias of −0.2 bpm for *f_R_* and 0.01 s for *T_tot_* as reported in [15,43]) as well as with those of non-contact technologies (e.g., LOAs of ±5 bpm and of ±3.4 bpm for *f_R_*, as reported in [22,44]). Considering all the estimated time-related respiratory parameters, we achieved comparable results in terms of median and IQR with those obtained in [24] (e.g., a median of 13.43 bpm in our work and a median of 16.0 bpm in Tamiya et al.). Considering the analysis of the respiratory-asynchrony-related parameters, the results show that the values of PA and PS estimated from signals retrieved from the video are quite comparable with those of the reference system. Considering that PA and PS values equal to 0° define synchronous signals while PA and PS of 180° indicate completely asynchronous ones [9], the results suggest that very slight asynchronies between compartments were observed.

The proposed non-contact system may be useful to investigate the occurrence of breathing discoordination between compartments more in depth, which could result in a phase shift in the related breathing signals. In addition, it can be used as an instrument for the continuous monitoring of real-time respiratory-related parameters to assess the health status of patients (e.g., hemiplegic subjects that may have paradoxical motion of the respiratory muscles, which can result in a breathing discoordination between compartments).

This study has proven the feasibility of using a single RGB digital camera to frame the anterior surface of the torso and retrieve the displacements of the thoracic and abdominal compartments expressed in mm independently. In addition, the proposed non-contact system is unobtrusive and does not create discomfort for the subjects. However, it is important to acknowledge certain limitations that should be considered when evaluating the potential application of the proposed non-contact system in a clinical setting. The applicability of our non-contact system depends on the subject being as motionless as possible during the assessment process. This may be difficult to achieve, especially in uncooperative patients or in subjects suffering from discomfort. Another constraint is that the proposed system’s testing was limited to a limited number of seated subjects in a dedicated room. Nevertheless, it is crucial to highlight that its complete non-invasive nature is a significant advantage, especially in situations where conventional contact-based methods may be impractical or carry potential risks. Further investigations will be devoted to a deeper analysis of PA and PS between compartments to evaluate the respiratory asynchronies and improve the accuracy of the non-contact system. With this aim in mind, tests on a larger number of volunteers and patients (e.g., hemiplegic patients) will be performed.

## Figures and Tables

**Table 1:** Errors in the estimation of the displacements of the four non-reflective markers (i.e., 24 mm, 22 mm, 20 mm, and18 mm) during the three simulated time intervals (i.e., t = 3 s, t = 1.5 s, and t = 1 s) at four imposed displacement—cov. disp—(i.e., d_1_ = 101 mm, d_2_ = 51 mm, d_3_ = 21 mm, and d_4_ = 11 mm).

Marker 1 (D = 24 mm)			
	Error (mm)		
cov. disp (mm)	t = 3 s	t = 1.5 s	t = 1 s
d_1_ = 101	−0.24	−0.16	−0.45
d_2_ = 51	1.21	0.73	0.75
d_3_ = 21	0.22	0.06	0.06
d_4_ = 11	0.44	−0.01	−0.01
**Marker 2 (D = 22 mm)**			
	**Error (mm)**		
**cov. disp (mm)**	**t = 3 s**	**t = 1.5 s**	**t = 1 s**
d_1_ = 101	−0.03	−0.27	−0.26
d_2_ = 51	0.70	0.68	0.86
d_3_ = 21	0.25	0.18	0.06
d_4_ = 11	0.37	0.18	0.12
**Marker 3 (D = 20.2 mm)**			
	**Error (mm)**		
**cov. disp (mm)**	**t = 3 s**	**t = 1.5 s**	**t = 1 s**
d_1_ = 101	0.14	−0.70	−0.40
d_2_ = 51	0.88	0.80	0.62
d_3_ = 21	−0.13	0.38	0.38
d_4_ = 11	−0.02	−0.01	−0.08
**Marker 4 (D = 18 mm)**			
	**Error (mm)**		
**cov. disp (mm)**	**t = 3 s**	**t = 1.5 s**	**t = 1 s**
d_1_ = 101	0.09	−0.80	−0.91
d_2_ = 51	0.86	0.24	0.28
d_3_ = 21	−0.12	−0.02	−0.02
d_4_ = 11	0.26	0.10	0

**Table 2:** Mean and standard deviation (SD) obtained for the time-related respiratory parameters (i.e., *f_R_*, *T_i_*, *T_e_*, and *T_tot_*) for each volunteer both for the reference system (OEP) and the proposed non-contact system.

Volunteer	Parameter	Mean ± SD	
		OEP	Proposed System
S1	*f_R_* [bpm]	13.93 ± 1.07	14.05 ± 1.44
	*T_i_* [s]	2.27 ± 0.12	2.22 ± 0.36
	*T_e_* [s]	2.09 ± 0.24	2.13 ± 0.43
	*T_tot_* [s]	4.33 ± 0.34	4.31 ± 0.44
S2	*f_R_* [bpm]	12.35 ± 2.63	12.35 ± 2.62
	*T_i_* [s]	1.92 ± 0.34	2.11 ± 0.44
	*T_e_* [s]	3.21 ± 0.91	3.01 ± 1.97
	*T_tot_* [s]	5.11 ± 1.20	5.11 ± 1.20
S3	*f_R_* [bpm]	10.65 ± 0.85	10.65 ± 0.78
	*T_i_* [s]	2.19 ± 0.18	2.49 ± 0.36
	*T_e_* [s]	3.51 ± 0.36	3.14 ± 0.49
	*T_tot_* [s]	5.67 ± 0.49	5.66 ± 0.45
S4	*f_R_* [bpm]	16.65 ± 6.28	15.56 ± 6.12
	*T_i_* [s]	1.61 ± 0.49	1.78 ± 0.65
	*T_e_* [s]	2.49 ± 1.24	2.65 ± 1.38
	*T_tot_* [s]	4.16 ± 1.65	4.48 ± 1.71

## Data Availability

The data presented in this study are available upon request from the corresponding author. The data are not publicly available due to privacy restrictions.
